# Extreme heat events heighten soil respiration

**DOI:** 10.1038/s41598-021-85764-8

**Published:** 2021-03-23

**Authors:** Hassan Anjileli, Laurie S. Huning, Hamed Moftakhari, Samaneh Ashraf, Ata Akbari Asanjan, Hamid Norouzi, Amir AghaKouchak

**Affiliations:** 1 Department of Civil and Environmental Engineering, University of California, Irvine, CA USA; 2 Department of Civil Engineering and Construction Engineering Management, California State University, Long Beach, CA USA; 3 Department of Civil, Construction and Environmental Engineering, University of Alabama, Tuscaloosa, AL USA; 4 Department of Building, Civil and Environmental Engineering, Concordia University, Montreal, Canada; 5 Universities Space Research Association, Mountain View, CA USA; 6 Department of Construction Management and Civil Engineering, New York City College of Technology, The City University of NY, New York, NY USA; 7 Department of Earth System Science, University of California, Irvine, CA USA

**Keywords:** Microbial communities, Climate sciences

## Abstract

In the wake of climate change, extreme events such as heatwaves are considered to be key players in the terrestrial biosphere. In the past decades, the frequency and severity of heatwaves have risen substantially, and they are projected to continue to intensify in the future. One key question is therefore: how do changes in extreme heatwaves affect the carbon cycle? Although soil respiration (Rs) is the second largest contributor to the carbon cycle, the impacts of heatwaves on Rs have not been fully understood. Using a unique set of continuous high frequency in-situ measurements from our field site, we characterize the relationship between Rs and heatwaves. We further compare the Rs response to heatwaves across ten additional sites spanning the contiguous United States (CONUS). Applying a probabilistic framework, we conclude that during heatwaves Rs rates increase significantly, on average, by ~ 26% relative to that of non-heatwave conditions over the CONUS. Since previous in-situ observations have not measured the Rs response to heatwaves (e.g., rate, amount) at the high frequency that we present here, the terrestrial feedback to the carbon cycle may be underestimated without capturing these high frequency extreme heatwave events.

## Introduction

Climate extreme events such as heatwaves are progressively playing a significant role in the regional and global terrestrial carbon cycle^1–4^. The amount and rate of the associated impacts are challenging to characterize^5^, due to a number of relatively infrequent direct observations and spatially limited in-situ experiments^6,7^. This leads to an incomplete understanding of the terrestrial carbon cycle^5^ and therefore, contributes to uncertainties in various climate models^8,9^. Several studies have focused on large-scale and low-temporal resolution relationships between the aboveground terrestrial biosphere (i.e., plants and trees) and heatwaves^6–8^. However, uncertainties and existing knowledge gaps are more pronounced when considering short-term responses of the terrestrial biosphere, in particular belowground microbial communities^10^, to extreme heat events, which we investigate here.

Soil respiration (Rs), the carbon dioxide (CO_2_) flux exhaled by plant-roots and microbes from the soil to the atmosphere^11^, is the second largest contributor to the global carbon cycle^12^. Rs releases almost 310 Gt CO_2_ per year^13^, which is nine times larger than anthropogenic CO_2_ emissions^14^. Factors such as soil organic matter, spatial characteristics and soil properties of the study site play roles in the rate of respiration^15^. Nevertheless, the primary controlling factors of Rs are soil temperature, soil moisture (SM), and substrate supply^16–18^. However, air temperature anomaly is significantly and positively correlated with change in Rs when SM is sufficient available^19^. Moreover, soils harbor three times more carbon than the Earth’s atmosphere and therefore, any minor changes in the behavior of the subsoil community (i.e., heterotrophs and autotrophs) due to changes in the temperature variability could have direct and immense impacts on the carbon cycle^20–23^.

Heatwaves may occur due to high pressure atmospheric conditions and an interaction between the dry soil surface and increasing air temperatures^24–27^. Furthermore, the frequency and severity of heatwaves have elevated globally over roughly the last seven decades, and future scenarios project an even more notable increase in the number of severe heatwaves^5,28,29^. Although definitions can vary, a heatwave is generally described as a period of ongoing extremely hot days and it can occur during the daytime and/or nighttime^30,31^. In the Data Analysis Section, we describe the more specific heatwave definition that we applied in our study. Heatwaves can change the Rs characteristics since biological systems are more vulnerable to extreme events, rather than the gradual increase in mean characteristics of the system, due to the short response time and amplified response strengths^2,5,17^. Heatwaves are unpredictable and considered to be pulse and press perturbations, because microbes and plant-roots are sensitive to interruptions in either their activities, decomposition, or both^13^. These perturbations could propagate the effects on Rs by a multiple compared to a gradual temperature increase. Heatwave impacts on the Rs dynamics (e.g., rate) between the land surface and atmosphere remain highly uncertain due to limited knowledge about the mechanisms controlling Rs^10,13,17,32^, especially in semi-arid areas where the interrelationship between SM and temperature plays a crucial role in the carbon cycle^11,33^.

Extreme heat manipulation experiments on soil respiration have been conducted in laboratories and field studies^34,35^. However, these extreme heat manipulation experiments on Rs mainly focus on a short period of time and do not provide insight into the natural ecosystem where multiple factors (e.g., hourly/daily temperature variability, SM content, and seasonal effects) influence Rs. Furthermore, previous laboratory experiments neither provide information about differences occurring during the day and night in an ecosystem nor do they shed light on the potential high frequency (e.g., hourly and sub-hourly) response of Rs to heatwaves.

**Figure 1:**
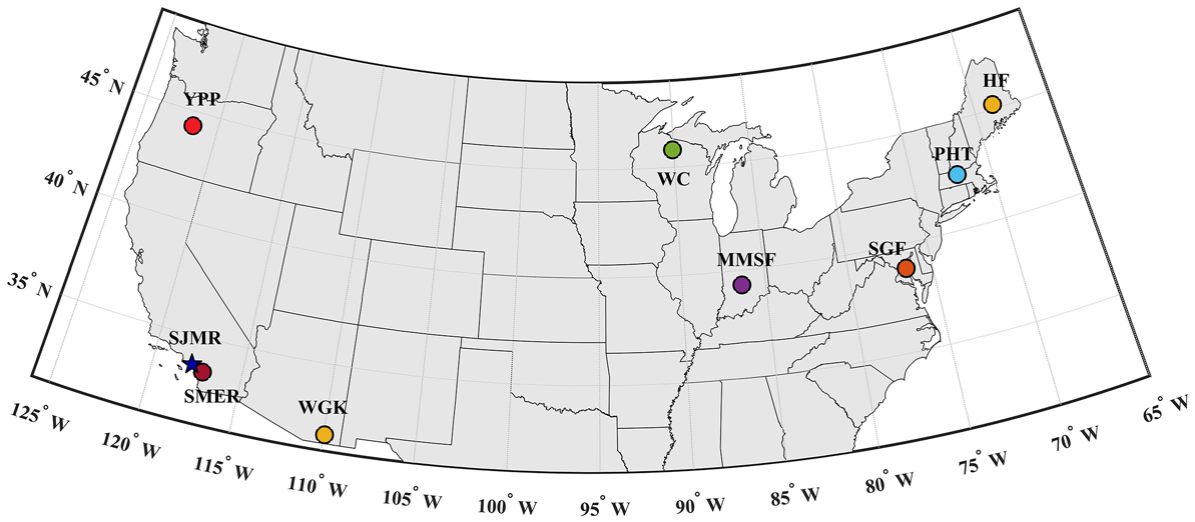
Study sites across the CONUS. Colors denote each study site and names (see Table 1) in which measurements have been observed. Zhang et al.^37^ (MMSF) performed two studies at the same location. Varner et al. (2010) and Savage et al.^38^ (PHT) gathered data at the same location, but during different time periods. This Figure was created with Matlab version 2019b.

Here, we present results from continuous high frequency (i.e., sub-hourly) in-situ observations of Rs that we collected, in addition, to Rs observations obtained at multiple sites across the contiguous United States (CONUS), to determine its response to heatwaves (see Fig. 1 for the location of the sites). We characterize the impacts of Rs under heatwave and non-heatwave conditions using a probabilistic model (see the Materials and Methods for details) and determine whether a significant difference between Rs during these two types of conditions occurs. We begin with a detailed description of the Rs response to heatwaves at the San Joaquin Marsh Reserve (SJMR) site. We then extend our framework and analysis to ten other sites, from the continuous soil respiration database (COSORE v0.5)^36^, across the CONUS to better understand how different soils and climatic characteristics impact such findings. Using a conditional multivariate model, we tested the hypothesis that heatwaves significantly change Rs. The main objective of this study is to answer the following questions: What is the likelihood that Rs changes during heatwaves? To what extent does heatwave impact Rs during dry and wet conditions?

## Materials and method

### Field measurements at the SJMR

In one location within the San Joaquin Marsh Reserve (SJMR), we collected original Rs data as shown in Fig. 1 marked with a star symbol. The measurement device was set up at an untouched area within SJMR, adjacent to the University of California, Irvine (33° 39ʹ 32.7ʺ N, 117° 50ʹ 55.9ʺ W). Overall, the region has a Mediterranean climate with an annual mean temperature and precipitation of ~ 17 °C and 300 mm, respectively^39^. The taxonomic classification of the soil at the study area is characterized as omni clay, Fine, montmorillonitic (calcareous), thermic Fluvaquentic Haplaquolls, with potential Hydrogen (pH) of 8.5, Soil Organic Matter (SOM) of 3.50%, carbon content of 2.03%, and calcium carbonate (CaCO_3_) of 3%^40,41^. The SM field capacity (FC) and permanent wilting point (PWP) for the site was reported to be 0.33 and 0.20 m^3^/m^3^, respectively^42^. In semi-arid areas such as at the SJMR site, Rs is also sensitive to SM content, particularly during dry periods/seasons^11^. Anjileli et al.^43^ concluded that SM at the SJMR varies between 0.18 and 0.37 m^3^/m^3^ and that Rs increases with precipitation events; however, when soil becomes saturated (beyond 0.33 m^3^/m^3^), Rs remains unresponsive to additional precipitation pulses.

The automated soil respiration (Rs) system LI-8100A from LI-COR (LI-8100A, LI-COR, Inc., Lincoln, Nebraska, USA) measured sub-hourly Rs at the study site day and night from Feb. 2016 till Feb. 2017. The LI-8100A is an Automated Soil Gas Flux System which measures CO_2_ flux from the soil using a single long-term transparent chamber and an Analyzer Control Unit (ACU). The infrared gas analyzer (IRGA) installed in the ACU measures the change in CO_2_ in the chamber. One polyvinyl chloride (PVC) collar with a diameter of 20.3 cm and height of 11 cm was inserted into the soil to a depth of 6 cm one week before measuring Rs to limit soil disturbance and to allow repeated measurements. The vegetation within the collar was cleared off to make sure that the soil remains bare over the entire observation period. We programmed five measurements per hour with an observation length of two minutes over a period of one year.

Near the chamber at 5 cm depth below the ground surface, the soil volumetric water content, also called SM, was measured using an ECH_2_O model EC-5 (Decagon Devices, Inc., Pullman, WA, USA). In addition, the system was powered with a 260 W solar panel. Overall, ~ 7% of the Rs data were not observed due to instrument failure. The limitations of the data include relatively short length of record and lack of multiple devices for analyzing spatial heterogeneity in the region.

### COSORE database

We obtained the open COSORE v0.5^36^ from the Ben Bond-Lamberty GitHub website (https://github.com/bpbond/cosore). In this study, we excluded study sites if they included manipulated soil plots (e.g., excavated trench, topsoil removed, pipes installed under the soil plots) and study sites in which a near weather station or long-term hourly air temperature data for heatwave analysis was unavailable. The study sites across the CONUS that we use from the COSORE v0.5 database are shown in Fig. 1. Specific site information, including latitude/longitude, land types, SM thresholds, and the nearest weather stations to the study area are listed in Table 1. Interested readers are directed to the corresponding publications listed in Table 1 for detailed information related to each observational study and the associated measurement methodologies employed.

**Table 1:** Study sites for continuous soil respiration observation obtained from COSORE.

Site name	Lat; Lon	Study Site; SM (m^3^/m^3^)	Land cover	Nearest weather station	Data source	Publication
SDSU Santa Margarita Ecological Reserve (SMER)	33.442; − 117.164	Dry < 0.12 Wet > 0.20	Open Shrubland	Camp Pendleton Mcas, CA	Mauritz and Lipson^44^	10.5194/bgd-10-6335-2013
San Joaquin Marsh Reserve (SJMR)	33.658; − 117.849	Dry < 0.23 Wet > 0.27	Wetland	Santa Ana John Wayne Airport, CA	Anjileli et al.^43^	10.1029/2018JG004640
SERC-GCReW Forest (SGF)	38.875; − 76.552	Wet > 0.45	Deciduous Broadleaf Forest	Camp Springs Andrews Afb, MD	Pennington et al.^45^	10.5194/bg-2019-218
Morgan Monroe State Forest (MMSF)	39.323; − 86.413	Dry < 0.25 Wet > 0.35	Deciduous Broadleaf Forest (Maple)	Indianapolis Intern. Airport, IN	Zhang et al.^37^	10.1016/j.agrformet.2018.05.005
Morgan Monroe State Forest (MMSF)	39.323; − 86.413	Dry < 0.30 Wet > 0.37	Deciduous Broadleaf Forest (Oak)	Indianapolis Inter. Airport, IN	Zhang et al.^37^	10.1016/j.agrformet.2018.05.005
Young Pine Plantation (YPP)	44.323; − 121.608	N. A	Evergreen Needleleaf Forest	Redmond Airport, OR	Ruehr et al.^46^	10.1016/j.agrformet.2012.05.015
Prospect Hill Tract (Harvard Forest) (PHT)	42.540; − 72.170	Dry < 0.30 Wet > 0.36	Deciduous Broadleaf Forest	Worcester, MA	Savage et al.^38^	10.1111/j.1365-2435.2008.01414.x
Prospect Hill Tract (Harvard Forest) (PHT)	42.540; − 72.170	N. A	Deciduous Broadleaf Forest	Worcester, MA	Phillips et al.^18^	10.1029/2008JG000858
Willow Creek (WC)	45.806; − 90.080	Dry < 0.24 Wet > 0.33	Deciduous Broadleaf Forest	Wausau Asos, WI	Phillips et al.^56^	10.5194/bg-10-7999-2013
Howland Forest (HF)	45.204; − 68.7402	Dry < 0.25 Wet > 0.29	Wetland	Millinocket Airport, ME	Sihi et al.^47^	10.1016/j.agrformet.2018.01.026
Walnut Gulch Kendall (WGK)	31.736; − 109.942	Dry < 0.06 Wet > 0.12	Grassland	Sierra Vista Airport, AZ	Roby et al.^48^	10.3390/soilsystems3010006

In order to define the SM threshold for wet and dry conditions of each study site, we consider values above the 70th quantile as wet (i.e., high SM) and below the 30th quantile as dry (i.e., low SM). The SM thresholds using in-situ observation and remote sensing data are listed in Table 1.

The hourly long-term historical air temperature data (i.e., at least 30 years of hourly air temperature data) from weather stations for each study area are obtained from the National Oceanic and Atmospheric Administration (NOAA) mapping tool website (https://gis.ncdc.noaa.gov/maps/ncei/cdo/hourly).

### Data analysis

It is expected that the frequency and severity of regional heatwaves increases globally over the next century^1,28,49^. A heatwave is generally characterized as a period of consecutive extremely hot days and it can occur during the daytime and/or nighttime^30^. Here, we define a heatwave using the 85th percentile of the long-term air temperature climatology (T_lgtrm_)^50^ for each hour of the month at each study site. This means that for each month we obtain 24 threshold values, over the long historic record. In order to obtain the heatwave thresholds, we use long-term hourly air temperature data, collected from the nearest weather station to the study site (see Table 1) from the National Oceanic and Atmosphere Administration (NOAA) website (https://gis.ncdc.noaa.gov/maps/ncei/cdo/hourly).

We averaged the measured sub-hourly Rs data collected into an hourly time series to match the temporal frequency of the air temperature described above. We also matched the data sets temporally to be compatible in the analysis, due to missing data in both data sets.

Previous studies have made relatively little effort to identify the likelihood of Rs given heatwaves by using high frequency observations. High frequency Rs data sets combined with probability density function (PDF) can yield new insights related to changes in the Rs distribution in response to shifts in drivers (e.g., heatwaves, SM)^43^. The PDF of the variable of interest (e.g., Rs) conditioned on either one parameter (e.g., heatwaves) or two parameters (e.g., heatwaves and SM) can be calculated by using equations (1) and (2), respectively^51^, as follows:

1 $${\text{f}}_{{{\text{Rs}}|{\text{HW}}}} \left( {r|{\text{h}}} \right) = [{\text{f}}_{{{\text{HW}}}} \left( h \right){ } \cap {\text{ f}}_{{{\text{Rs}}}} \left( r \right)]/{\text{f}}_{{{\text{HW}}}} \left( h \right)$$

2 $${\text{f}}_{{{\text{Rs}}|{\text{HW}},{\text{SM}}}} \left( {r|{\text{h}},m} \right) = [{\text{f}}_{{{\text{HW}}}} \left( h \right){ } \cap {\text{ f}}_{{{\text{Rs}}}} \left( r \right){ } \cap {\text{ f}}_{{{\text{SM}}}} \left( m \right)]/[{\text{f}}_{{{\text{HW}}}} \left( h \right){ } \cap {\text{ f}}_{{{\text{SM}}}} \left( m \right)]$$

where ${\text{f}}_{{{\text{Rs}}}} \left( r \right)$, ${\text{f}}_{{{\text{HW}}}} \left( h \right)$, and ${\text{f}}_{{{\text{SM}}}} \left( m \right)$ represent the marginal PDF of Rs, heatwaves, and SM respectively. To display the impact of heatwave conditions on Rs, we use the exceedance probability, which describes the likelihood of Rs exceeding a given threshold (${\text{Rs}}$ > $r$) for heatwave/non-heatwave (HW = ${h}_{1}$, ${h}_{2}$, …) conditions and soil moisture (SM = ${m}_{1}$, ${m}_{2}$, …) conditions. For our exceedance probability analysis, the threshold corresponds to the mean Rs value for each site and the exceedance probability indicates the likelihood that the incident Rs is larger than its mean value^52^. For instance, the SJMR site threshold corresponds to ~ 1.1 μmol CO_2_/m^2^s. We require a minimum of 50 data points to implement PDF analyses for each given heatwave, non-heatwave, and SM cases. Please note that during heatwave/non-heatwave events, we use all Rs data (i.e., from the beginning to the end) that exceeds/fall below the 85th of the T_lgtrm_.

Furthermore, we use the two-sample Kolmogorov–Smirnov (KS) test, which is a built-in function in Matlab, to analyze and compare the distribution of heatwaves and non-heatwaves. The test reveals whether the data from the different events come from the same distribution at a significance level of 0.05 or 95% confidence interval.

## Results

**Figure 2:**
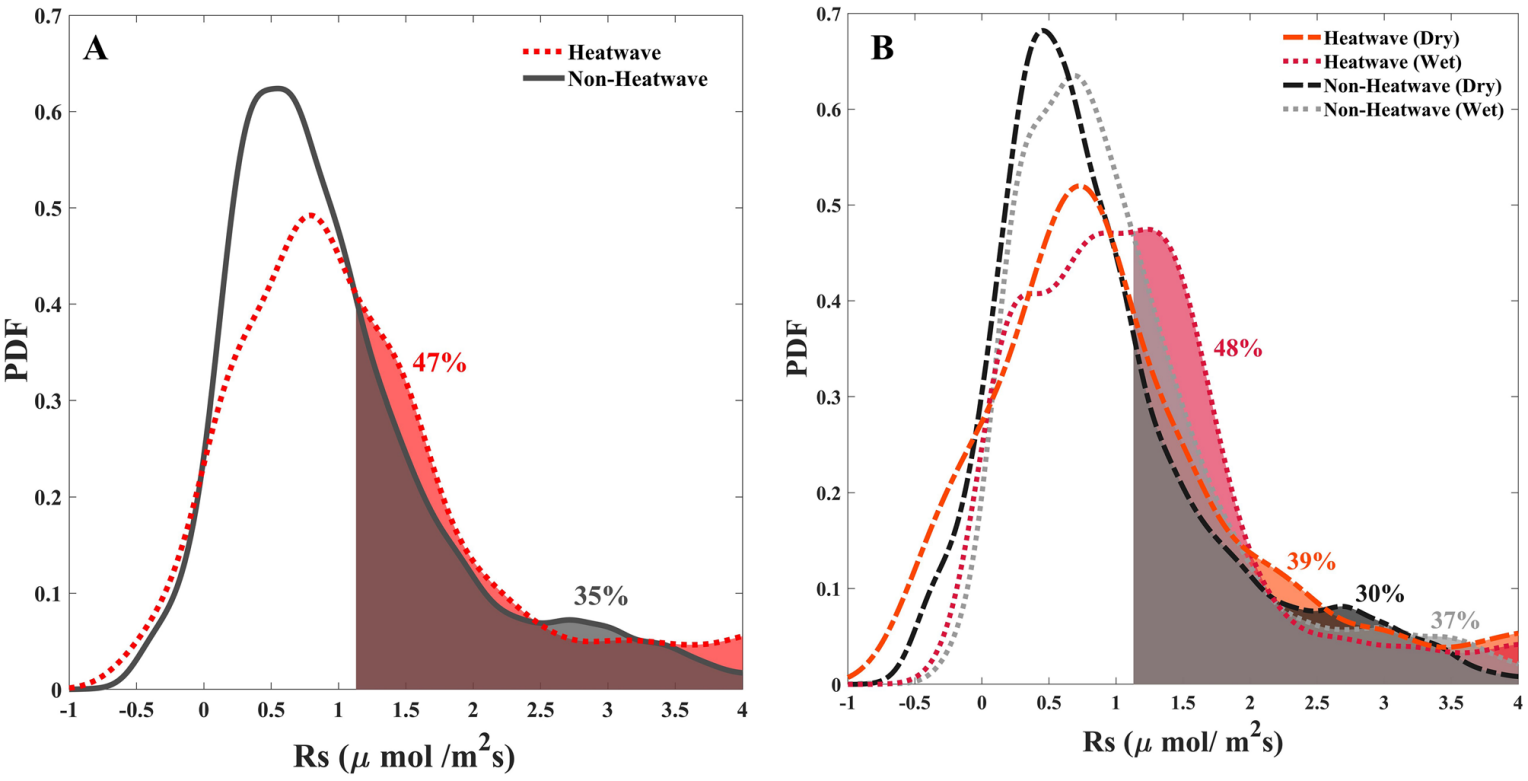
Probability density functions (PDFs) of soil respiration (Rs) for the San Joaquin Marsh Reserve (SJMR) over the study period February 2016 to February 2017. Shaded areas denote soil respiration exceeding the average soil respiration and the percentages indicate the corresponding exceedance likelihoods. (**A**) PDFs of soil respiration given heatwave (red dotted line) and non-heatwave conditions (black solid line). (**B**) PDF conditioned on heatwave/non-heatwave and two soil moisture regimes (i.e., dry and wet conditions).

Using the Kolmogorov–Smirnov (KS) test, we find that the distribution of Rs during heatwave periods at the semi-arid SJMR study site is significantly different than its distribution under non-heatwave conditions, at a significance level of 0.05 (or 95% confidence level) (see materials and methods section). Based on this detectable difference, we further compare the probability density function (PDF) of measured Rs conditioned on (1) heatwave and (2) non-heatwave conditions. The shaded area under the PDF curves in Fig. 2A corresponds to the likelihood of Rs exceeding its mean value (~ 1.1 μmol CO_2_/m^2^s) based on equation (1) in the Materials and Methods Section. The likelihood of Rs exceeding its mean value rises from 35% during non-heatwave cases to 47% during heatwave situations, which indicates a significantly higher likelihood of observing large Rs rates during heatwave events. Furthermore, the mode (i.e., most likely value) of the Rs distribution during heatwaves shifted to higher Rs rates with respect to its distribution during non-heatwave periods.

Figure 2B shows the distribution of Rs conditioned on heatwave/non-heatwave and dry/wet SM content scenarios based on equation (2) (see Materials and Methods). The shaded area under the PDFs indicates the likelihood of Rs surpassing its mean value during heatwave/non-heatwave periods conditioned on dry (dHW/dNHW) and wet (wHW/wNHW) SM cases. The results suggest a significantly higher likelihood of high Rs rates during heatwaves with low (dHW: 39%) and high (wHW: 48%) SM content compared to non-heatwave conditions with low (dNHW: 30%) and high (wNHW: 37%) SM content. The lower values of Rs during both dry conditions (dHW and dNHW) with respect to the wet condition (wHW and wNHW) cases can be attributed to limited SM availability^34,53^. Interestingly, we find that even for low SM content the occurrence of heatwaves leads to an increased likelihood of higher Rs rates (dNHW: 30% vs. dHW: 39%). Nevertheless, sufficient moisture availability during wHW and wNHW increases the likelihood of above average Rs by a factor of 1.3 with respect to both dry scenarios. As a result, when heatwaves impact Rs in a semi-arid area, we have, on average, 30% more Rs. Furthermore, we can confirm that SM is the key component dominating the response dynamics of Rs.

**Figure 3:**
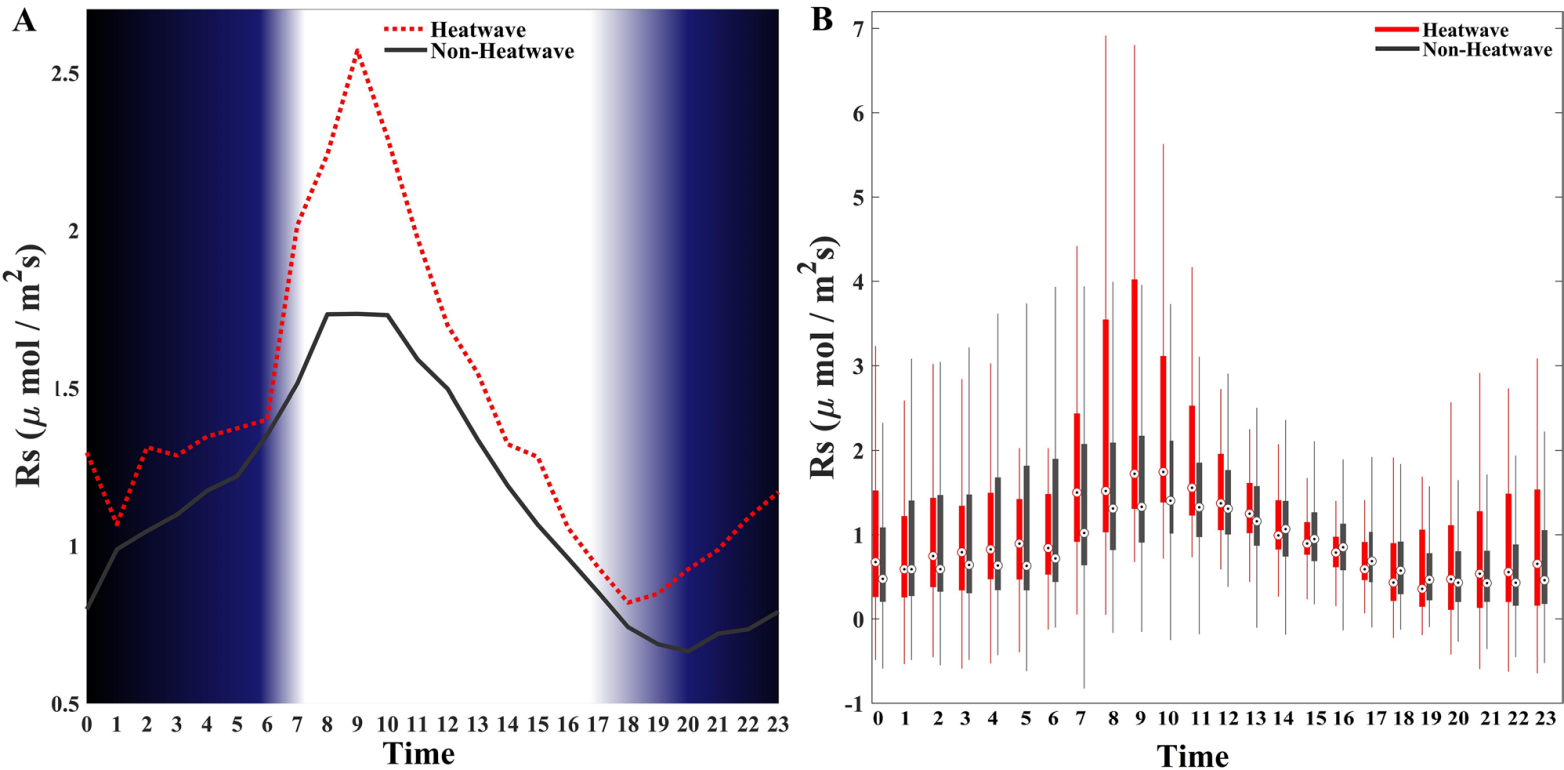
Mean diel Rs cycle and variability during heatwave and non-heatwave conditions at the San Joaquin Marsh Reservoir (SJMR) using the long-term hourly mean across all months. Local time (UTC-8 h) is shown. (**A**) Gradients in the shading denote dawn (5:45–7:15) and dusk (17:00–20:00). (**B**) Boxplots of Rs under heatwave (red) and non-heatwave (black) conditions. Each pair of boxplots represent the range of observed values for one hour of the day (in local time) under the various conditions, where the bottom (top) edge of the box indicates the 25th (75th) percentile and the circles demarcate the median.

Since Rs rates often vary on a diel basis (i.e., a 24-h continuous period, including both the day and night) due to hydroclimate and light variability, we explore how heatwaves change the diel variation of Rs (e.g., magnitude and diurnal pattern). In Fig. 3A, we provide a detailed, high-resolution description of the impacts of heatwave/non-heatwave events on the diel variation of Rs. The dotted/solid line in Fig. 3A shows the diurnal cycle of Rs or in other words the average Rs released under heatwave/non-heatwave conditions for each hour of the day. The results indicate that higher rates of soil respiration consistently occur during heatwaves as compared to non-heatwave conditions. Nonetheless, both climatic conditions exhibit the highest Rs rates between 7:00 and 11:00. Here and after, all times are provided in local time (UTC—8 h). Interestingly, we observe that during dusk between 17:00 and 20:00 (shown as a gradient in the shading), Rs increases gradually until dawn rather than maintaining an almost constant value (close to zero) throughout the night as previous studies have generally shown. Using dew point temperature information, Anjileli et al.^43^ concluded that an additional source of moisture (i.e. dew) other than precipitation arises after dusk (through dawn), which is responsible for the partial increase of Rs we observe^54,55^. Figure 3B displays the variability of heatwave/non-heatwave scenarios, associated with the results climatological mean diurnal cycles from Fig. 3A. The greatest impact of heatwaves on the Rs rate and its variability occurs during the morning (07:00–11:00) compared to non-heatwave conditions.

**Figure 4:**
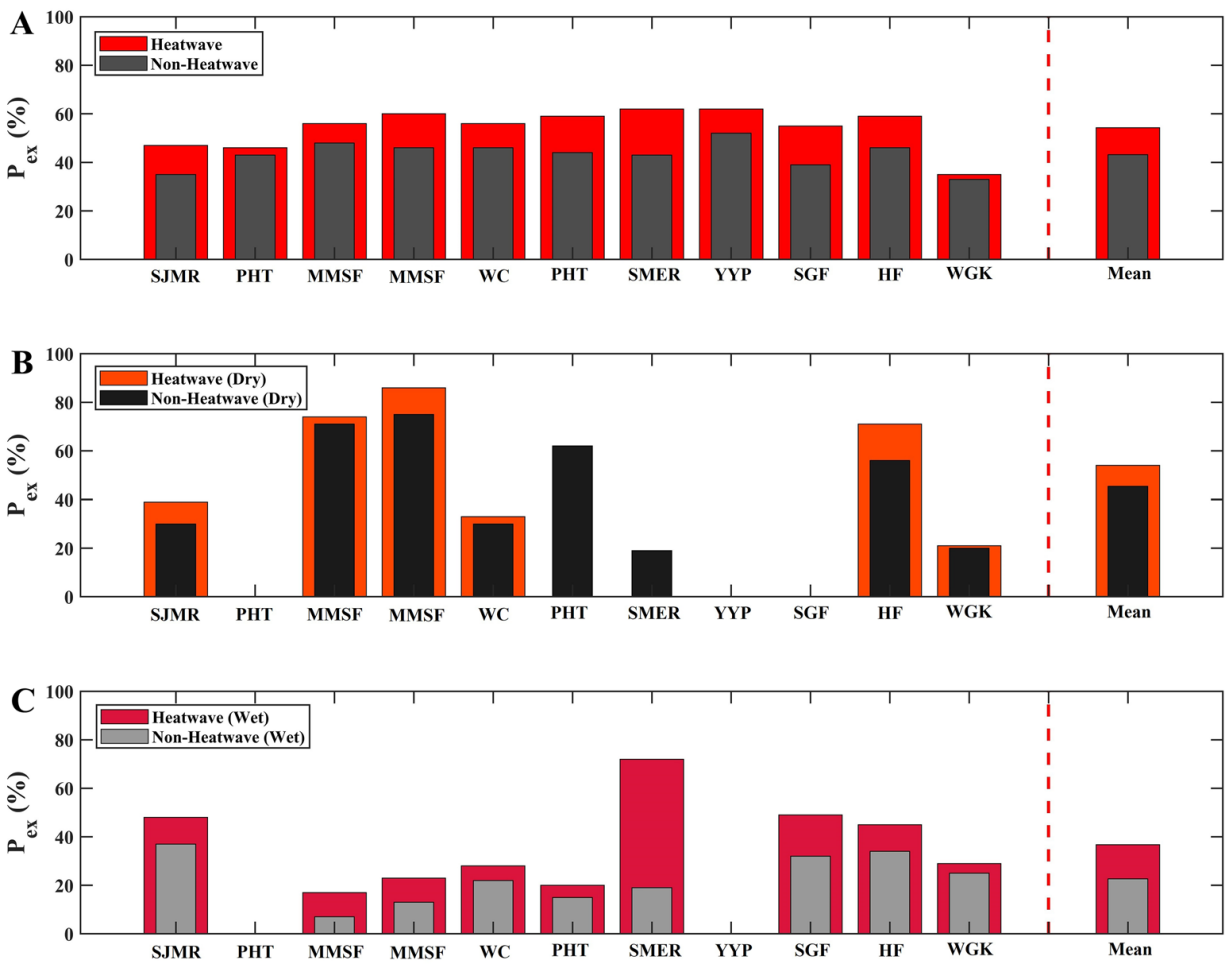
P_ex_ (%) indicates the corresponding exceedance likelihoods when exceeding its average soil respiration rate. The mean (right side beyond the dashed red lines) represents the average P_ex_ (%) of all eleven study sites. (**A**) P_ex_ (%) indicates soil respiration given heatwave and non-heatwave. (**B**) and (**C**) represents P_ex_ (%) of soil respiration given heatwave and non-heatwave and two soil moisture regimes (i.e., dry and wet condition), respectively. Due to insufficient data points at a given soil moisture regime (e.g., SMER, PHT, and SGF), the P_ex_ (%) have been excluded. The PHT and YPP study areas are not shown since soil moisture data was not available. In addition, the SGF study site displayed only measurement during the winter period; therefore, dry condition data was not available.

**Figure 5:**
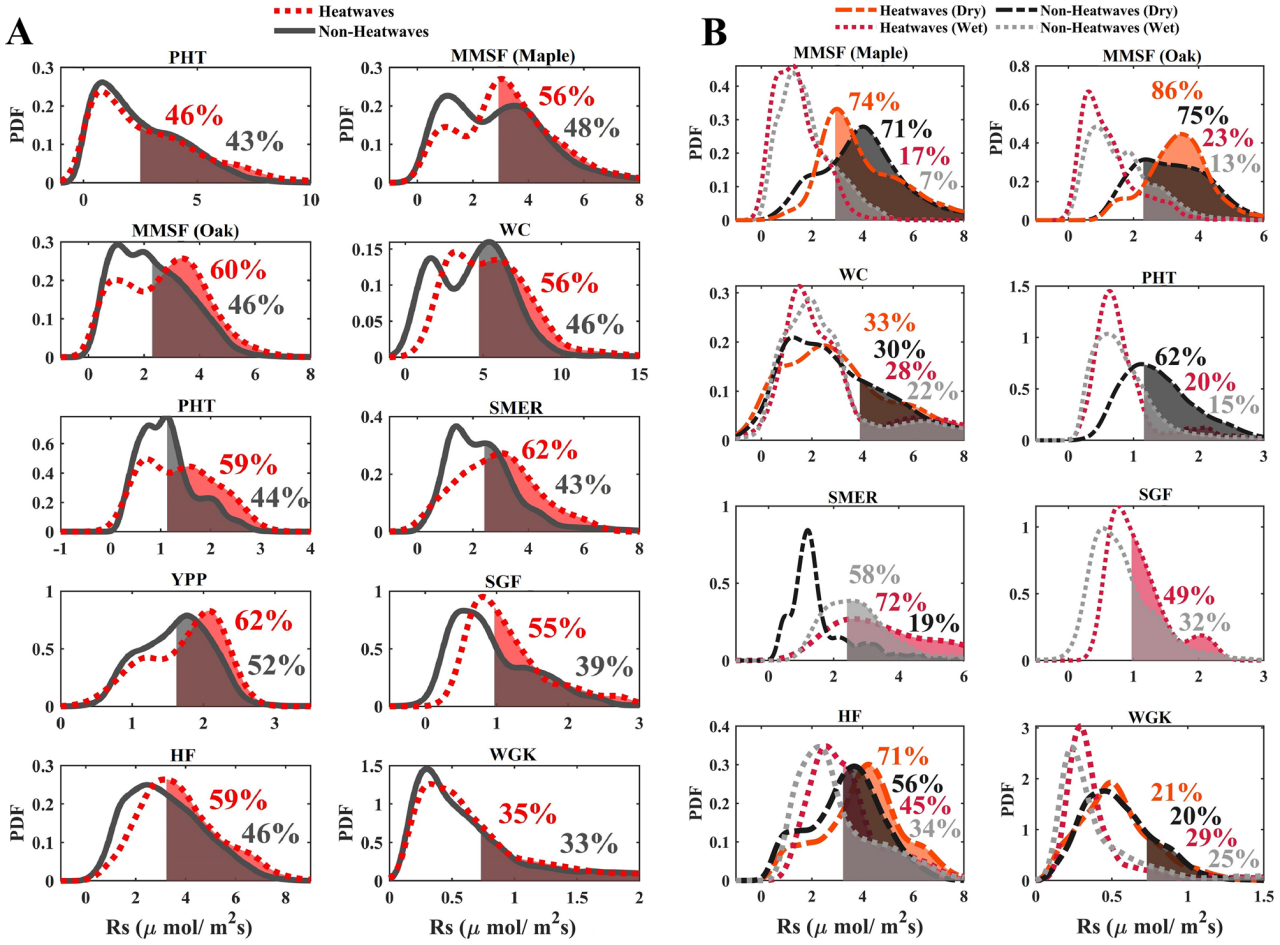
PDFs of soil respiration for the eight individual study sites. (**A**) PDFs of soil respiration given heatwaves and non-heatwaves. Shaded areas denote soil respiration exceeding its average rate and the percentages indicate the corresponding exceedance likelihoods. (**B**) PDF conditioned on heatwave/non-heatwave and two soil moisture regimes (i.e., dry and wet conditions). Some PDFs are excluded from the panels due to lack of data points, which if used may lead to a misrepresentation of the underlying relationships (see Materials and Methods).

We extend the above analysis to the additional ten sites across the CONUS for which in addition to Rs we had access to hourly temperature data for heatwave analysis^18,37,38,44–48,56^ (see Table 1 and Fig. 1), we find similar Rs responses to heatwaves across all sites. Each panel in Fig. 4 displays the probability of exceeding the Rs mean values [P_ex_ (%)]. The names of the study sites correspond to the author that has obtained the data (see Table 1). For a more detailed description of the conditional PDFs and their exceedance values, please see Fig. 5. Across the study regions, the P_ex_ during non-heatwaves elevates, on average, from 43 to 54% during heatwaves. Therefore, during heatwaves the Rs rate is 26% more compared to non-heatwaves across all study regions. The lowest P_ex_ value during non-heatwaves (35%) is found in the semi-arid study site (SJMR). Semi-arid areas are well known for the lowest Rs values due to their limited SM content^43^. We observe that the highest P_ex_ values occur during heatwaves (62%) in open shrublands (Mauritz) and an evergreen needleleaf forest (Ruehr) in California and Oregon, respectively.

Since Rs is sensitive to SM, we also investigate the Rs dynamics of the other study areas during heatwave and non-heatwave events conditioned on both dry and wet SM conditions as we show in Fig. 4B,C. Overall, we again find consistent results among the SJMR and the other sites in CONUS (cf. Figs. 2B, 5B). By averaging the same events and conditions of all study sites which is shown as the mean (beyond the dashed red lines) (i.e., wHW, wNHW, dHW, dNHW), we find that the P_ex_ during heatwave and wet conditions shows higher values (wHW: 36%) with respect to non-heatwave and wet conditions (wNHW: 21%). Performing the same analysis, we also show that during heatwave and dry events the P_ex_ increases (dHW: 54%) compared to non-heatwave and dry situations (dNHW: 45%). These results show that SM not only plays a key role in semi-arid areas, but also in other land cover regions.

## Discussion and conclusion

Although semi-arid regions have the lowest Rs rates compared to other ecosystems on earth^57^, our designed observational study and the study sites across the CONUS show that Rs responds to heatwaves and in particular, to concurrent heatwave and high SM conditions. It appears that microbial communities in soils are resilient to extreme heat events which temporarily leads to a significant increase in their respiration rates^17^. Schimel et al.^58^ concluded that microbial communities which have been exposed to perturbations earlier are usually more resistant to forthcoming disturbances compared with those that have not. This is particularly crucial since disturbances such as heatwaves are likely to increase in frequency and intensity in the next several decade^28^. On the other hand, climate change influences the nutritional balance (i.e. nutrient stoichiometry) of soils which has a crucial impact on the dynamics of the soil microbial community^59^. Therefore, the carbon cycle is biologically coupled from the molecular to global scales^60^.

Despite the fact that the complexity, diversity, and heterogeneity of the soil is intricate, multiple taxonomic and functional facets of diversity could describe the soil respiration response to heat extreme events ^10,17^. Even though the underlying processes still remain unclear, our results show that heatwaves increase the soil respiration by ~ 200 g CO_2_/m^2^ yr (by ~ 26% more than that of non-heatwave events) across the selected areas in the CONUS. These designed measurements across the CONUS are single-point measurements collected at different time periods ranging from 6 months to 3 years. However, they show a clear pattern of rising Rs rates during heatwaves. Most previous studies have focused on long-term changes (e.g., Rs response to change in mean temperature) and did not focus on short-term extremes like heatwaves. We argue that considering short-term extremes like heatwaves is critical when investigating CO_2_ flux feedbacks to the carbon cycle in a warming climate, particularly because heatwaves are project to increase in frequency and severity in the future. The ~ 200 g CO_2_/m^2^ yr change in Rs is based on 11 study sites across the CONUS and more in-depth research is needed to fully understand the contribution of extreme events such as heatwaves to Rs across the globe. Furthermore, this Rs amount could increase with longer and more severe heatwaves as expected in the future^1^. Given that the previous in-situ observations have not considered the Rs response to heatwaves (e.g., rate, amount), the terrestrial feedback to the carbon cycle may be largely underestimated without capturing the effects of heatwave events (and possibly other short-term extreme events).
